# Conditional deletion of *RB1* in the Tie2 lineage leads to aortic valve regurgitation

**DOI:** 10.1371/journal.pone.0190623

**Published:** 2018-01-05

**Authors:** Marina Freytsis, Lauren Baugh, Zhiyi Liu, Irene Georgakoudi, Philip W. Hinds, Lauren D. Black, Gordon S. Huggins

**Affiliations:** 1 Sackler School of Graduate Biomedical Sciences, Tufts University, Boston, MA, United States of America; 2 Molecular Cardiology Research Institute, Center for Translational Genomics and Cardiology Division, Tufts Medical Center, Boston, MA, United States of America; 3 Department of Developmental, Molecular and Chemical Biology, Tufts University School of Medicine, Boston, MA, United States of America; 4 Biomedical Engineering, Tufts University, Medford, MA, United States of America; Rutgers University Newark, UNITED STATES

## Abstract

**Objective:**

Aortic valve disease is a complex process characterized by valve interstitial cell activation, disruption of the extracellular matrix culminating in valve mineralization occurring over many years. We explored the function of the retinoblastoma protein (pRb) in aortic valve disease, given its critical role in mesenchymal cell differentiation including bone development and mineralization.

**Approach and results:**

We generated a mouse model of conditional pRb knockout (cKO) in the aortic valve regulated by Tie2-Cre-mediated excision of floxed *RB1* alleles. Aged pRb cKO animals showed significantly more aortic valve regurgitation by echocardiography compared to pRb het control animals. The pRb cKO aortic valves had increased leaflet thickness without increased cellular proliferation. Histologic studies demonstrated intense α-SMA expression in pRb cKO leaflets associated with disorganized extracellular matrix and increased leaflet stiffness. The pRb cKO mice also showed increased circulating cytokine levels.

**Conclusions:**

Our studies demonstrate that pRb loss in the Tie2-lineage that includes aortic valve interstitial cells is sufficient to cause age-dependent aortic valve dysfunction.

## Introduction

Aortic valve disease is a complex disease process characterized by progressive thickening and fibrosis of the aortic valve (AoV) leaflets leading to valve sclerosis, a precursor to valve mineralization and often frank bone formation that can cause restriction and/or regurgitation of blood flow from the heart. Statistics from 2017 indicate that more than 100,000 patients received an aortic valve replacement [1] and the prevalence of aortic valve disease increases markedly with age [2]. There is an important need to determine the molecular mechanisms contributing to aortic valve disease because the only treatment for advanced disease is surgical replacement [3] as medical therapy has not been found effective at slowing disease progression [4, 5].

During embryonic heart development, cells derived from the endocardium [6], the secondary heart field, and the neural crest [7] can be found in the fibrosa, spongiosa, and ventricularis layers of the AoV [8]. Following valve development, the valve interstitial cells (VICs) predominantly have an endothelial origin, [9] likely the result of endothelial-to-mesenchymal transformation of endocardial cells. Further supporting an endothelial origin of VICs, Tie2 lineage tracing studies produce strong staining of the aortic valve leaflet [8, 10, 11]. With age, the adult AoV increasingly includes CD45-positive cells derived from the hematopoietic system [12] that would also be expected to be derived from the Tie2 lineage [13]. Though normally quiescent, VICs can become activated, and differentiate into myofibroblasts expressing alpha smooth muscle actin (α-SMA) [14].

VICs produce an array of precisely oriented structural matrix proteins necessary for the leaflet to bear the load during diastole, while also sufficiently flexible to impart negligible outflow resistance during systole [15, 16]. In the diseased valve activated VICs deposit and reorganize the extracellular matrix (ECM) [17–19] leading to rearrangement of collagen fibers in the fibrosa and proteoglycan changes in the spongiosa layers [20, 21]. ECM disarray precedes development of CAVD, particularly in the aorta-facing fibrosa where calcific nodule and bone formation first appear [2, 22, 23].

The retinoblastoma protein (pRb) regulates two key factors relevant to aortic valve disease: maintenance of mesenchymal cell differentiation as well as bone formation and soft tissue calcification [24–27]. Given the mesenchymal nature of VICs and the appearance of osteogenesis in diseased human valves, we hypothesized that VICs deficient in pRb would lose their quiescent phenotype (they would become activated), leading to aortic valve dysfunction possibly resulting in leaflet mineralization. To interrogate this hypothesis we created a mouse model of targeted pRb loss using Tie2 directed cre recombinase expression because many of the VICs in the AoV leaflets are derived from endothelial cells [8, 9] and Tie-2-cre effectively deletes genes in endothelial-cell-derived VICs resident in the AoV leaflets [8, 10, 28]. Our results demonstrate that pRb in the Tie2 lineage is necessary to maintain aortic valve structure and function with age.

## Materials and methods

### Animals

The flox19-RB1 (*RB1*^fl/fl^) mice [29] were maintained in a C57BL/6 background. *RB1*^*fl*/fl^ females were bred to Tie2-Cre males, purchased from Jackson laboratories (B6.Cg-Tg (Tek-cre)1Ywa/J). *RB1*^fl/fl^;Tie2-Cre^-^ and *RB1*^fl/+^;Tie2-Cre^+^ from the first cross were bred to generate *RB1*^fl/fl^;Tie2-Cre^+^ (pRb cKO) and *RB1*^fl/+^Tie2-Cre^+^ (pRb het) mice for our experiments. The use of pRb het mice as controls is justified given the lack of observed phenotype. Genotype was determined by PCR analysis of genomic DNA from mouse tails. Genomic DNA was isolated and amplified by PCR using the following primers: *RB1*: 5’-GGC GTG TGC CAT CAA TG-3’ (forward primer) and 5’-AAC TCA AGG GAG ACC TG-3’ (reverse primer); Cre: 5’-GTG AAA CAG CAT TGC TGT CAC TT-3’ (forward primer) and 5’-GTG AAA CAG CAT TGC TGT CAC TT-3’ (reverse primer).

For further verification of Tie2 activity, Tie2-Cre mice were bred to B6 mice bearing the targeted insertion of a floxed(stop)CAG-TdTomato allele into the ROSA26 locus (strain AI9, JAX#007909), such that a red fluorescent protein variant (tdTomato) is seen where Cre is expressed. Tissues from 2–4 month old Ai9;Tie2Cre mice were used for histological evaluation of recombination based on TdTomato fluorescence.

A total of 39 mice (15 experimental mice and 24 control mice) were used for these studies. 25 mice died for experimental endpoints and 9 mice died because in the opinion of the Division of Laboratory Animal Medicine and the investigators euthanasia was required for humane endpoints. 5 animals died without obvious cause or antecedent illness. Humane endpoints were defined as follows: Any animals displaying signs of distress including hunched posture, piloerection, labored breathing, tumors exceeding 5mm in any dimension, inability to eat or drink, or loss of 10% or more of body weight will be sacrificed within 24hrs. Aged mice with detectable valve dysfunction were watched closely and euthanized if they showed signs of edema or tachypnea with decreased cage movement that would be consistent with congestive heart failure. Animals were monitored 3 times per week and 24hours post echocardiograph procedures. Researchers handling mice went through mandatory animal handling training at Tufts University for proper evaluation of mouse health. Mice received water and food ad libitum, housed in cohorts of not more than 5 adults, and were otherwise maintained in accordance with a protocol approved by the Tufts University Institutional Animal Care and Use Committee (Protocol #: B2013-42 and B2016-11).

### Echocardiography

In vivo valve structure and function were evaluated at 2 and up to 12 months of age for all animals, and a subset at 6 months, using an ultra-high frequency, high-resolution ultrasound (Vevo2100; VisualSonics, Inc., Toronto, ON, Canada). The chests of the mice were treated with a chemical hair remover to reduce ultrasound attenuation. Mice were anesthetized with 1–2% isofluorane inhalation, and placed on a heated platform to maintain temperature during the analysis. Two-dimensional imaging was recorded with a 40 MHz transducer to capture long-axis projections with guided B-Mode and color and pulsed-wave Doppler. Doppler interrogation was performed on the aortic valve outflow in the parasternal long-axis view to assess stenosis and regurgitation using a sample volume toggle to optimize the angle of interrogation. A modified right parasternal long-axis view was required in some cases to ensure ascertainment of the maximum velocity. Color flow Doppler echocardiography, in which flow movement toward the transducer is shown in red and that away from the transducer is shown in blue, was applied at sampling points indicated in the 2-dimensional images from a long-axis view. Measurements of ventricular function and peak gradient were calculated using the integrated software of the Vevo1200. Aortic regurgitation was defined by retrograde blood flow across the aortic valve into the left ventricular outflow tract for more than half of the diastolic period identified by Doppler echocardiography and by color-flow Doppler video. ImageJ was used to calculate aortic root diameter in diastole from B-mode stills, with 3 cycles averaged per animal.

### Blood pressure measurement

Blood pressure was measured in normal diet fed mice aged 10–12 months by tail cuff plethysmography using the Kenda Coda System. Mice were acclimated and trained for 2–3 days by 20 tail cuff inflations. For the following 2 days, blood pressure measurements were recorded and averaged for each mouse. At least 3 mice from each sex and genotype were used for this analysis.

### Histology

Hearts from 2-month-old and aged animals were harvested, rinsed well in cold PBS, and fixed for up to 24 hours in 10% neutral buffered formalin. Fixed hearts were submitted to the Jackson Laboratories Histology lab for processing, paraffin embedding, and sectioning. The tissues were processed overnight on a sakura tissue-tek VIP tissue processor. Blocks were grossly trimmed for correct orientation and embedded in paraffin. Blocks were faced to appropriate area (3 leaflets present) using 4x objective on microscope. 5um thick sections were taken of area 3–4 sections per slide. H&E stained slides were stained on Leica Autostainer XL and the Pentachromes were done using American Mastertech "Russel-Movat" Pentachrome kit. Images of the Movat stained valves were also taken with a Nikon 800E microscope using a 10x objective. Histological images from six Rb het and six cKO were analyzed to look at GAG composition as a percentage of total valve area. The images were first processed with Adobe Photoshop CC 2015 to isolate the valve, removing background blood and other debris from the images. Next, the RGB images were broken into the corresponding red, green, and blue images so that the Movat stain for GAGs (light blue) could be isolated. Using Cell Profiler [30], the images were inverted and the red and green channels were combined (in a 1:1.1 ratio) and the blue channel (multiplied 1.4 times) was subtracted from the result. This resulted in an image that was used to isolate the GAG area using a manual threshold. The total area of the valve was found using a manual threshold of the green channel and was used to calculate the GAG percentage area for each sample image.

### Immunohistochemistry

Sections were deparaffinized and rehydrated in graded ethanol washes. After blocking in 5% normal serum or BSA, sections were incubated overnight at 4°C with the following antibodies: anti-α-SMA (Abcam ab5694,1:1000) and anti-phospho-histone H3 (Cell Signaling #9701, 1:100). Alexfluor-conjugated anti-rabbit secondary antibodies were used to detect anti-pH3. Slides were mounted in Vectashield mounting media with DAPI (Vectorlabs H-1200) and visualized with fluorescence microscopy. HRP-conjugated anti-rabbit secondary antibodies were used to detect anti-α-SMA, with subsequent DAB-based colorimetric detection. A no-primary antibody control was used for each antibody to detect false-positives and autofluoresence. TUNEL staining was performed using the ApopTag Fluorescein In Situ Apoptosis Detection Kit (EMD Millipore S7111), per manufacturer’s instructions. Immunofluorescent stained sections were imaged on a Nikon 800E epifluorescent microscope with a Spot RT2 digital camera. Image analysis and quantification were performed in CellProfiler [30]. The α-SMA stained images were taken using a Nikon 800E microscope using a 40x objective. Images were taken of the full valve (all three leaflets) and edited in Adobe Photoshop CC 2015 to remove any remnants of the aorta, starting at the root of each leaflet. CellProfiler was used to determine the area of the valve leaflets and the area of positive α-SMA staining in order to find the activated percentage area of each valve.

### Atomic force microscopy

Valve tissue stiffness was measured using atomic force microscopy (AFM), with a Veeco Dimension 3100 AFM with Novascan borosilicate glass particle probes. We used probe tips (10 μm diameter bead) with a rated spring constant value of 0.6 N/m and the Hertzian theory was used to calculate the Young’s Modulus. The Young’s Modulus was calculated using a MATLAB code for each indent curve over an entire 2D force volume and then averaging these values for each sample. From these measurements, we were able to calculate changes in valve tissue stiffness between groups.

### Second harmonic generation imaging

Second harmonic generation signal from collagen fibers was collected using a Leica TCS SP2 microscope equipped with a Ti:Sapphire laser which provided 800 nm excitation, dry Leica 20x/0.7 NA objective, and 400 nm ± 10 nm (ET400/20X) filter. At least 2, 3D volumes of valve leaflet were analyzed for each sample valve (n = 3 for both pRb het and cKO valves). Fiber orientation and variance were determined using a previously described method that relies on a weighted vector summation approach. The two-dimensional variance is a metric of the collagen fiber alignment that has a value of one for completely disorganized fibers and zero for all fibers within a field exactly aligned in the same direction [31].

The SHG images were also used a metric of fibrosis. After masking images for SHG signal only, image stacks were analyzed to find the average pixel intensity, as a measure of collagen fibrosis [32], per image. The stack of images was averaged to find a single value for each location. The data was then normalized to one.

### LC-MS/MS proteomics

Individual aortic valve leaflets were excised from flash frozen hearts; the three aortic leaflets from a single animal were collected and pooled to generate one sample; three aortic samples per genotype (for a total of six animals; three of each type) were analyzed and weighed (all measured at 0.01mg of tissue for each sample). Valve leaflets were lyophilized for six hours before undergoing a urea digested at 4°C using a stir bar to agitate the sample. Sample protein was then collected with an acetone precipitation [33]. Samples were then frozen before being sent to Beth Israel Deaconess Medical Center Mass Spectroscopy Core Facility for liquid chromatography–tandem mass spectroscopy (LC-MS/MS) analysis. Trypsin was used to digest the protein prior to analysis. Spectra with a 95% confidence were kept for analysis; there was an average spectral count of 690 for each sample and a total of 395 identified proteins. Resulting spectral counts were used to quantify abundance of cellular proteins and ECM proteins then individual counts were normalized by total spectral counts of cellular and ECM proteins collected per sample. ECM and cellular proteins with fold changes of either greater than 1.5 times or less than 0.6 times for the pRb het samples compared to the pRb cKO were included in a principle component analysis (PCA) with functional data (stiffness measurements and variance quantification) collected previously on the leaflets. PCA was done in SigmaPlot (Systat Software, San Jose, CA) using a correlation matrix and average eigenvalues for the components. The component scores and component loadings were plotted using MATLAB. The component scores were grouped using the *k*-means cluster algorithm (*k* = 2), as previously described [34, 35], in MATLAB; the loading scores were also grouped using k-means clustering (*k* = 4). Both algorithms were iterated 100 times to help assure convergence of the cluster centroids. ECM proteins were classified using the MatrixDB [36].

### Serum collection and analysis

Blood was collected after an overnight fast by cardiac puncture immediately after euthanasia. Serum was collected after clotting and centrifugation, the stored at -80°C. Cytokines were analyzed by a multiplexed ELISA array (Aushon BioSystems, Inc; Mouse Cytokine 1 CiraPlex™ Array) on Cirascan™ immunoassay system (Aushon BioSystems, Inc.)

### Statistical analysis

Aortic regurgitation incidence was evaluated with a two-tailed Fisher’s exact test. Other metrics were tested with an unpaired, two tailed Student’s T-test. Differences were considered significant at P-value <0.05.

## Results

### Deletion of pRb in murine aortic valve leaflets produces aortic valve regurgitation

**Fig 1:**
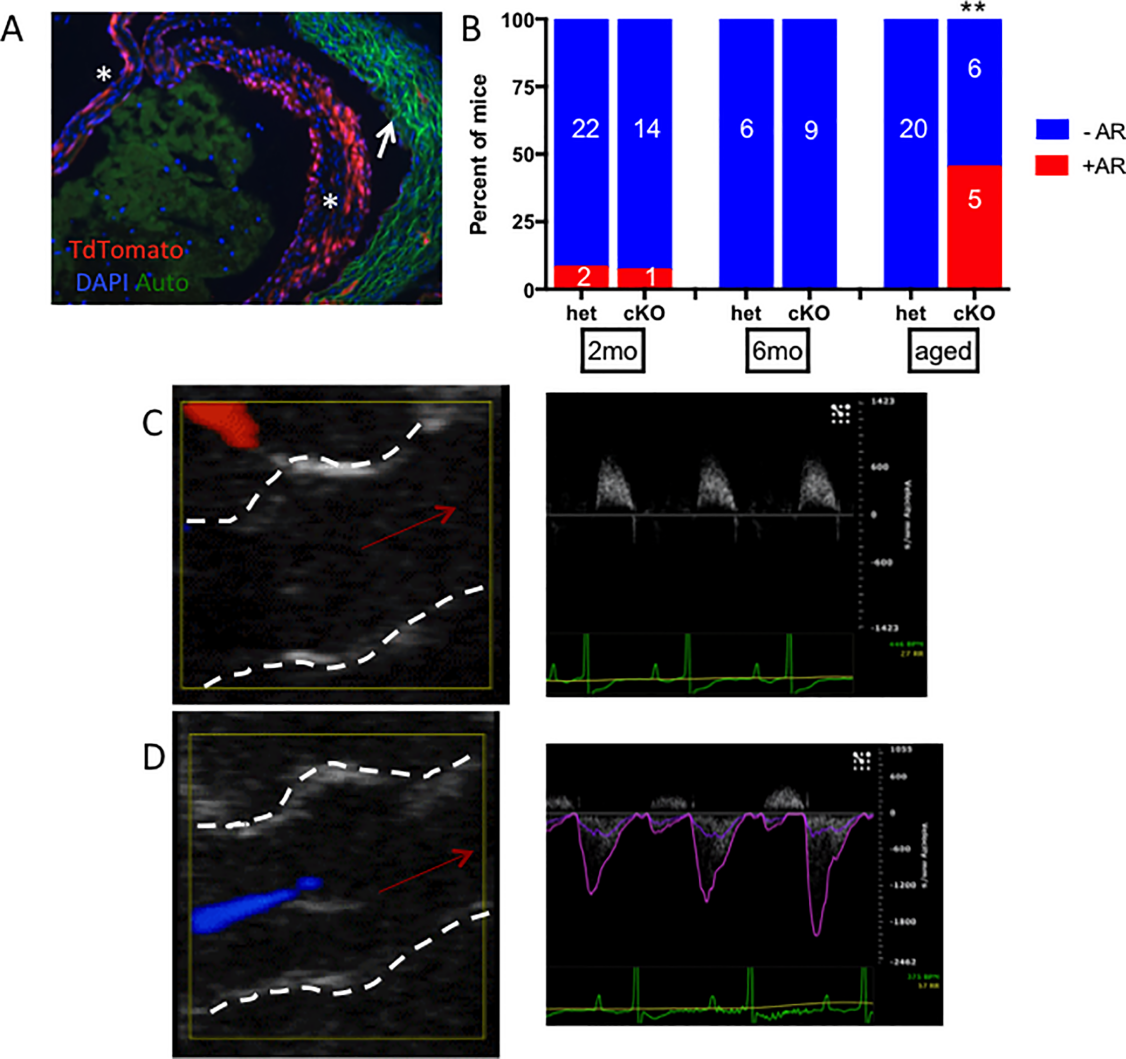
Tie2-driven loss of pRb causes aortic regurgitation (AR) with age. A) Confirmation of Tie2Cre-activity within leaflet of Ai9 mouse, where recombination throughout the leaflets (indicated by *) indicated by TdTomato fluorescence. Autofluorescence is seen in the aortic wall (indicated by arrow) B) Incidence of AR, as evaluated with Doppler by a blinded analyst, with age (**, Fischer’s exact test p = 0.0027). “Aged” indicates 10–12 month old animal. Sex did not have an effect on regurgitation incidence within the pRb cKO group (p = 0.55). C-D) Representative images are presented of color Doppler during diastole (left panel, left ventricular and aortic root structures indicated by dashed white line and direction of blood flow indicated by red arrow) and Doppler flow velocity tracing recorded from the left ventricular outflow tract (right panel). C) 2-month old animal. D) Aged pRb cKO with regurgitation: regurgitant flow at the aortic valve was recorded as a blue flow in the left ventricle during diastole in addition to retrograde diastolic Doppler flow recorded from the ventricular outflow tract.

Because whole-body *RB1* knockout mice die in utero [37], we utilized a conditional knockout model strategy whereby mice with loxP-flanked exon 19 of *RB1* (*RB1*^*fl/fl*^) were crossed with Tie2-Cre mice to delete pRb from the aortic VICs, as well as other endothelial-derived tissues [11]. Tie2 activity in the majority of VICs throughout the aortic leaflets was confirmed by TdTomato expression in Tie2-Cre; Ai9 mice (Fig 1A). Mice homozygous for *RB1*^fl/fl^ and heterozygous for the Tie2-Cre transgene are designated conditional pRb knockout (pRb cKO) mice while littermate *RB1*^+/fl^;Tie2-Cre^+^ mice were used as heterozygous (pRb het) controls. pRb cKO mice were viable and demonstrated a Mendelian ratio at weaning (χ^2^ P value = 0.86). Our experimental protocol designated echocardiographic and histologic analysis at one year of age, though more pRb cKO than het mice died or were sacrificed for humane reasons before that time (2 pRb het vs. 7 pRb cKO mice; S1 Fig). The reason for premature death in pRb cKO mice is not precisely known; however, Tie2-Cre also drives recombination in hematopoietic stem cells [13], and pRb-deficiency in hematopoietic cells can cause myeloproliferative disease [38, 39].

We aged 24 pRb het animals and 15 pRb cKO animals; at two and six months the aortic valve function was normal in pRb cKO and het animals by echocardiography. At 10–12 months of age (referred to as “aged” mice throughout the manuscript), 5 of 11 living pRb cKO mice were found to have severe aortic valve regurgitation (AR) identifiable by color flow Doppler (Fig 1B and 1D), while AR was not observed in 20 aged pRb het mice (Fig 1C; Table 1). Three additional pRb cKO mice had mild or trace AR. The mean ± SD peak velocity of the regurgitant jet was -2113 ±1967 mm/s in pRb cKO mice. The aortic root diameters measured by B-mode echocardiography during systole in pRb cKO and het animals were similar (Table 1), consistent with AR not being secondary to dilation of the aortic root. Functional aortic valve stenosis was not found in any animal during our study. pRb cKO heart weights normalized to tibia length were significantly heavier than pRb het hearts, suggesting mild left ventricular hypertrophy with preserved contractile function (Table 2). We considered whether increased afterload might explain the AR; however, tail cuff blood pressures in pRb cKO and het mice were similar (Table 2).

**Table 1:** Hemodynamic evaluation and aortic root diameter of aged mice.

	pRb Het		pRb cKO	
	Female (n)	Male (n)	Female (n)	Male (n)
**Peak velocity (mm/s)**	1205 ±86 (6)	1426 ±158 (6)	1206 ±38 (4)	1482 ±94 (8)
**Peak gradient (mmHg)**	5.96 ±0.83 (6)	8.64 ±1.63 (6)	5.83 ±0.36 (4)	8.99 ±1.17 (8)
**AoV Root Diameter (mm)**	1.17 ±0.03 (11)	1.22 ±0.03 (10)	1.22 ±0.05 (5)	1.28 ±0.05 (7)

**Table 2:** Evaluation of heart function, weight, and systemic blood pressure in aged mice.

	pRb Het		pRb cKO	
	Female (n)	Male (n)	Female (n)	Male (n)
**Heart Rate (beats/min)**	702 ± 52 (3)	595 ± 20 (5)	615 ± 55 (4)	609 ±72 (4)
**EF (%)**	66.38 ±5.39 (3)	60.28 ±3.88 (5)	68.16 ±7.74 (4)	59.38 ±3.66 (4)
**FS (%)**	36.25 ±3.89 (3)	32.14 ±2.73 (5)	38.62 ±5.61 (4)	31.46 ±2.43 (4)
**Systolic BP**	112.73 ±7.06 (3)	134.83 ±6.74 (5)	115.23 ±4.81 (4)	122.21 ±2.85 (4)
**Diastolic BP**	78.05 ±4.43 (3)	95.60 ±3.19 (5)	80.96 ±2.95 (4)	84.20 ±3.54 (4)
**Normalized Heart Weight (mg/mm)**	87.11 ±3.5 (8)	119.2 ±5.2 (7)	112.8 ±10.8 * (4)	147.3 ±5.8 ** (7)

EF, ejection fraction; FS, fractional shortening; BP, blood pressure. Heart weight normalized to tibia length. Values shown are mean ± SD. T-test:

*P-value = 0.016,

**P-value = 0.0036;

sex-matched cKO compared to Het.

### Loss of pRb causes ECM remodeling and VIC activation

**Fig 2:**
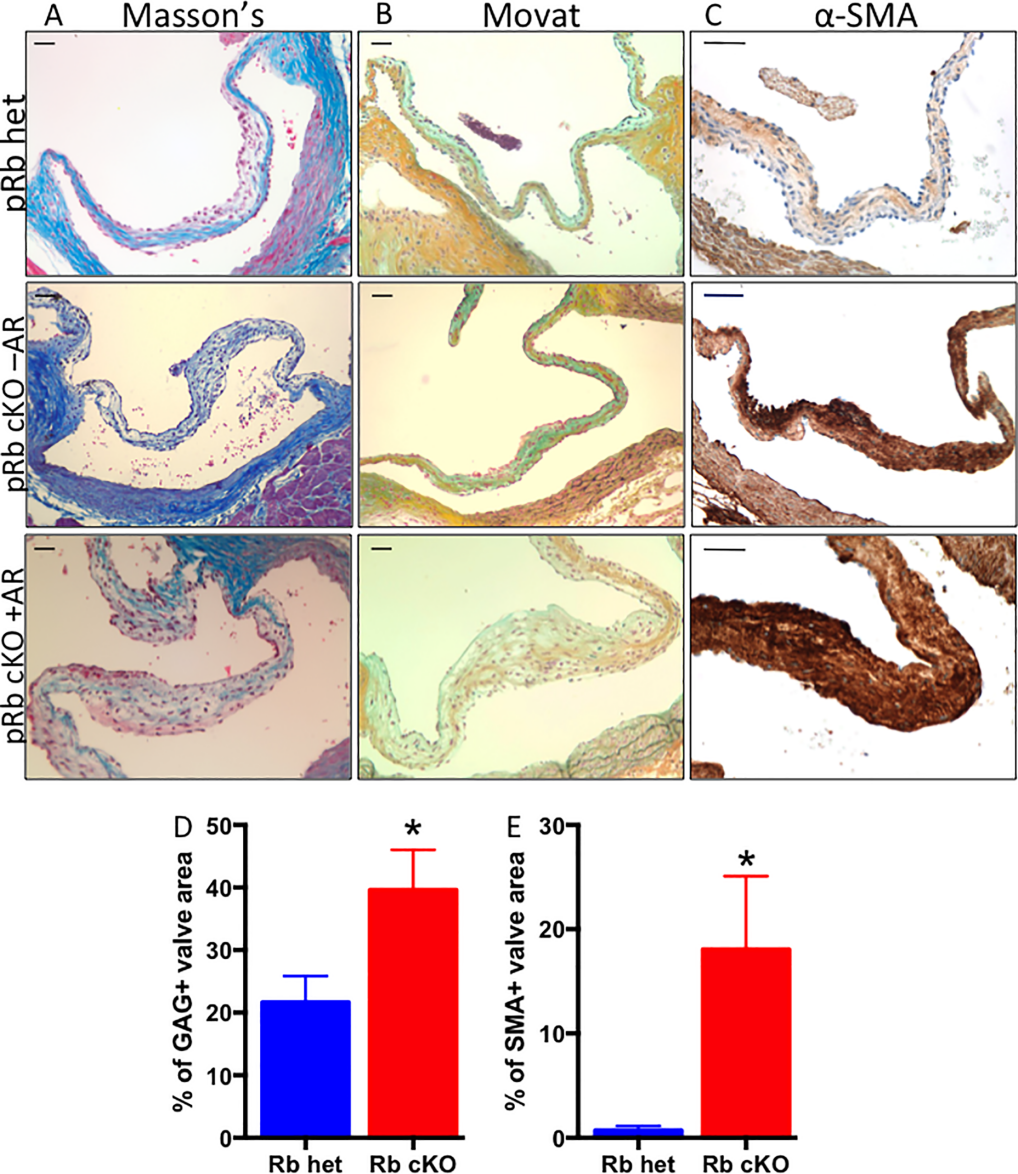
Representative histology of aortic valve leaflets from aged mice demonstrates changes in pRb cKO AoV. A) Masson’s trichrome showing reduced collagen staining (blue) in leaflet from pRb cKO mouse with aortic regurgitation (AR). B) Movat pentachrome showing more diffuse collage staining (yellow) in fibrosa, but normal proteoglycan staining (blue) in the spongiosa layer of the leaflet from pRb cKO with AR. C) Immunohistochemistry for α-SMA, demonstrating presence of activated myofibroblasts throughout leaflets of pRb cKO mouse with and without AR. Scale bar is 50μm. D) Quantification of the percent area of the valve expressing GAGs using a Movat stain. Mean ± SEM; Student T-test P-value = 0.04. E) Quantification of α-SMA, represented as percent of valve area with positive staining. Mean ± SEM; Student T-test P-value = 0.02.

We next analyzed fixed heart sections using histological stains to determine if morphological changes are present in the pRb cKO aortic valves. Aortic root and valve leaflet calcification were not observed by Alizarin Red and Von Kossa staining (S2 Fig). Masson’s Trichrome staining showed a more diffuse or weak collagen staining pattern in pRb cKO valves with AR (Fig 2A). Additionally, Movat pentachrome stain showed changes within the collagenous fibrosa layer of pRb cKO valve leaflets; cKO valves had a significantly higher GAG content (Fig 2B and 2D). In every pRb cKO valve, from mice with or without aortic valve regurgitation, intense α-SMA staining was observed throughout the leaflets compared with pRb het leaflets (n = 7 het, 3 cKO with AR, 4 cKO without AR; Fig 2C). By comparison, aortic valve leaflets from 2-month-old pRb cKO mice showed a pattern of histological and α-SMA staining similar to het mice, consistent with the changes in valve architecture at 10–12 months being associated with aging (S3 Fig).

### pRb cKO aortic valve leaflets show similar proliferation and apoptosis

**Fig 3:**
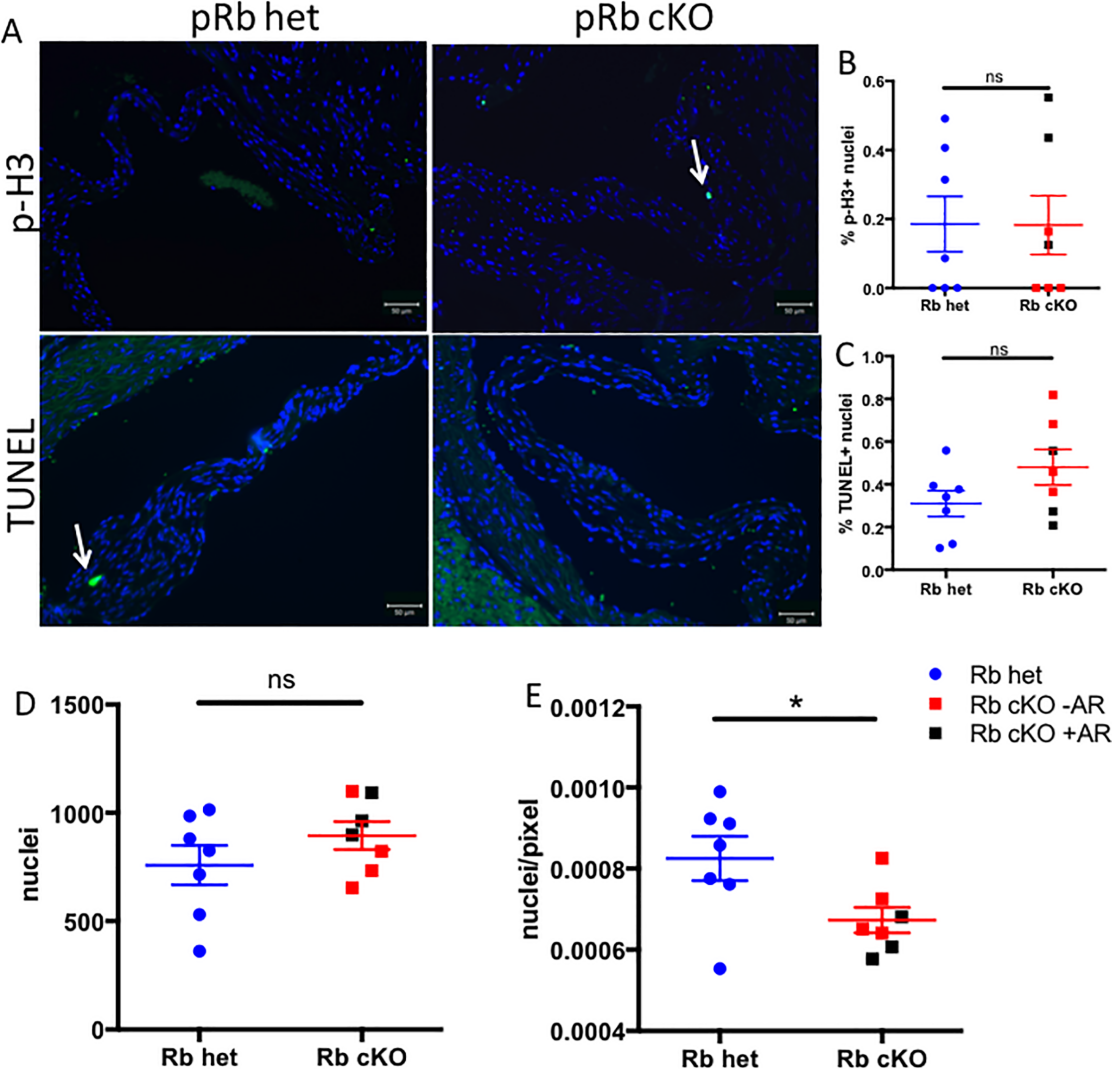
Aged pRb cKO aortic valves do not have increased proliferation or apoptosis. A) Representative staining for phospho histone H3 (p-H3; top panels, green) and TUNEL (bottom panels, green). Nuclei are stained blue with DAPI. B) Proliferation, shown as p-H3 expression, was not significantly different between genotypes. C) Apoptosis, as measure by TUNEL staining, was not significantly different between genotypes. D) Absolute number of nuclei per valve were not significantly different, but cellular density (E), expressed as DAPI+ nuclei per pixel of aortic valve leaflet, was significantly lower in pRb cKO valves (Student’s T-Test P = 0.03). n = 7 per genotype, mean+/-SEM.

Given the well-established role for pRb in cell cycle regulation [40] and because VIC proliferation and apoptosis have been observed in diseased human aortic valves [41], we asked whether differences in proliferation and cell death were found in the pRb cKO aortic valves. Cellular proliferation, measured by phospho-histone H3 expression (p-H3; Fig 3A and 3B), and apoptosis, measured by TdT-mediated dUTP-biotin nick end labeling (TUNEL; Fig 3A and 3C) were infrequently and similarly observed in both groups. Thus, differences in cell cycle regulation do not explain valve dysfunction in the pRb cKO mice. Furthermore, there was no significant difference in the number of DAPI-positive nuclei within the valve leaflets (Fig 3D). Interestingly, the nuclear density, assessed by the number of DAPI-positive nuclei normalized to cross-sectional leaflet area, was significantly lower in pRb cKO AoV leaflets than controls (Fig 3E). These results are consistent with expansion and remodeling of the extracellular matrix rather than cellular proliferation being responsible for pRb cKO aortic valve thickening.

### pRb cKO valve leaflets have disordered matrix and increased stiffness

**Fig 4:**
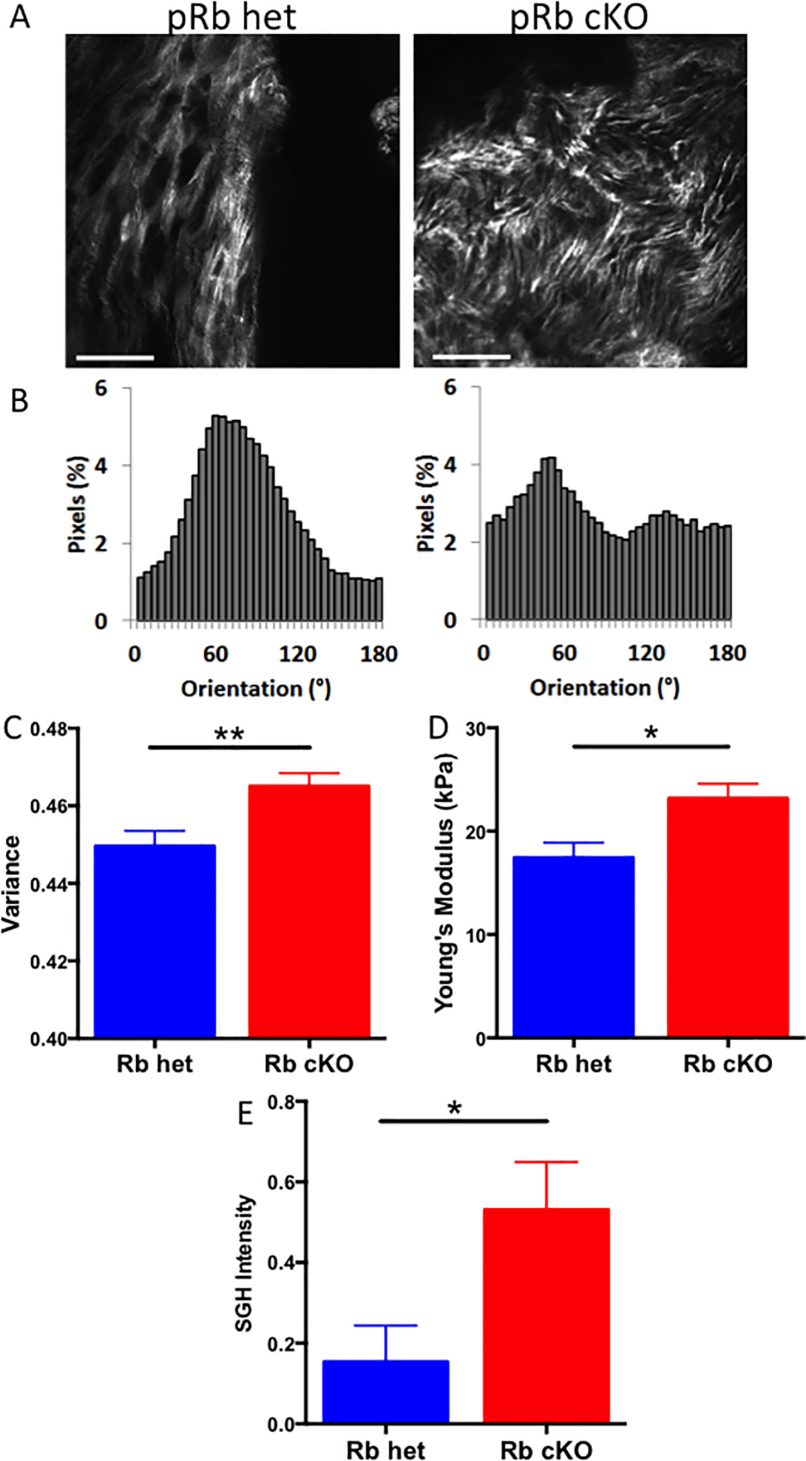
Structural analysis of AoV leaflets reveals differences in stiffness and collagen organization between pRb het and cKO mice. A) Representative second harmonic generation (SHG) images of mouse AoV leaflets. These images were used to calculate the variance of collagen fiber angle with representative fiber orientation data shown in B) and total variance data shown in C) (n = 3; Student’s T-test P = 0.003). D) Atomic force microscopy (AFM) was used to determine the Young’s modulus of the AoV leaflets (n = 4) pRb het and 6 pRb cKO; Student’s T-test P = 0.02). E) Quantification of valve tissue fibrosis using SHG pixel intensity. Mean ± SEM; Student T-test P-value = 0.03. Scale bar is 50μm.

We next asked whether the increased leaflet thickening observed in pRb cKO mice was associated with structural changes in the ECM using two non-destructive microscopy techniques. Second harmonic generation (SHG) analysis of collagen fiber orientation demonstrated more variable collagen structural organization in leaflets from pRb cKO mice compared with control animals (Fig 4A). Collagen disorganization in experimental animals was further demonstrated by quantitative analysis of pixel-wise fiber orientation (Fig 4B), which revealed a bimodal distribution in the pRb cKO AoV leaflets compared with a unimodal distribution of the pRb het leaflets. The 2D variance, a metric of the overall collagen fiber alignment was significantly increased in pRb cKO valves, corresponding to increased fiber disorganization (Fig 4C). Next, using atomic force microscopy (AFM) we found that pRb cKO valves have a higher Young’s Modulus than controls consistent with increased leaflet stiffness (pRb het valves 17.44 ± 3.8kPa versus cKO valves 23.18 ± 3.1kPa (Fig 4D)). Greater SHG intensity further indicated that the Rb cKO valves are more fibrotic than Rb het valves (Fig 4E). Taken together, these results demonstrate that the aortic valve thickening observed in pRb cKO aortic valves is associated with a loss of the normally ordered array of valve collagen and ECM resulting in a stiffer valve leaflet.

### Loss of pRb in VICs results in structural changes in the ECM of aortic valve leaflets

**Fig 5:**
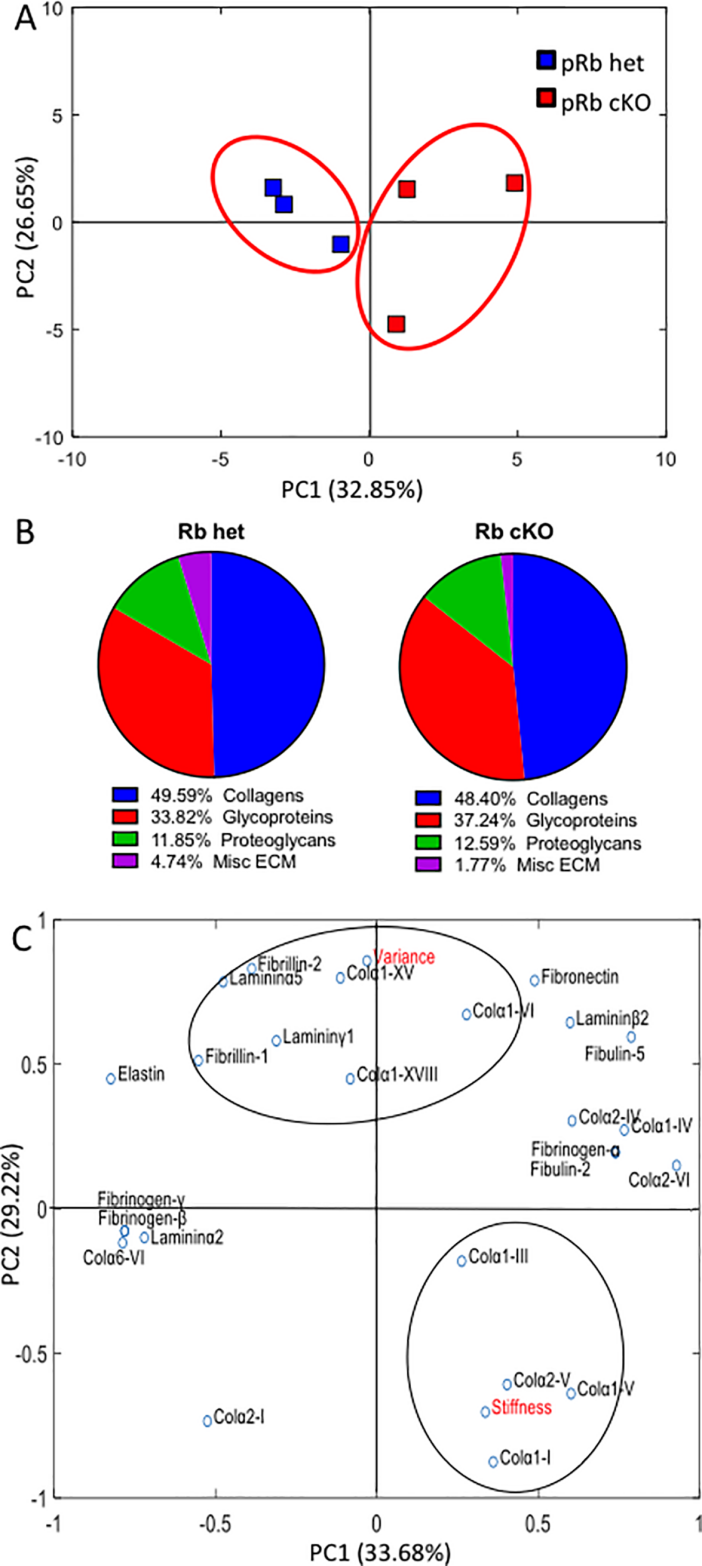
Analysis of the aortic valve proteome. Data represented as protein spectral counts normalized to total ECM and cellular protein spectral counts in samples from LC/MS/MS analysis of trypsin-digested valve leaflet tissue. pRb het were compared to pRb cKO mice with AR and grouped using k-means clustering. A) Principal Component Analysis (PCA) of all ECM and cellular protein counts that show substantial up (greater than 1.5 times) or down (less than 0.6) regulation of pRb cKO leaflets as compared to het controls, demonstrating clustering of both sample types. B) ECM composition breakdown of aortic valve leaflets describes the relative percentages of the matrix proteins in pRb cKO leaflets compared to the pRb het mice. C) Loading scores plotted for a PCA combining measured functional data (in red) with structural ECM proteins. Assuming 4 groups, *k*-means clustering was use to assess the relationships between the functional data and the proteomics output to look for ECM components that varied with the functional data outcomes. N = 3 per genotype.

To better characterize ECM changes in cKO valves we performed proteomic analysis on pRb het and cKO aortic valve leaflets. Proteomic analysis of the leaflets revealed a diverse group of proteins present in both the het controls and pRb cKO valves. Using principle component analysis (PCA) we created component scores for each valve sample based on proteins that were found in pRb cKO and het control valves. The PCA scores show distinct clustering of the pRb het valves and more varied distribution of the pRb cKO valves (Fig 5A), consistent with heterogeneous changes in the proteome caused by the loss of pRb. Additionally, sorting by ECM class shows minor variation in protein composition (Fig 5B). Individual normalized protein spectral counts from collected LC MS/MS data and statistical comparisons of the data can be found in S4 Fig. Lastly, to correlate the direct effect of structural ECM components with the measured leaflet functional data, we performed a second PCA and plotted loading scores of the structural ECM components with the stiffness and variance functional data derived from the SHG and AFM analysis, respectively (Fig 5C). These findings demonstrate that the functional measurements of stiffness and ECM variance clustered with ECM components important for tissue mechanics or matrix remodeling, respectively.

### Increased cytokine levels in aged pRb cKO mice

**Fig 6:**
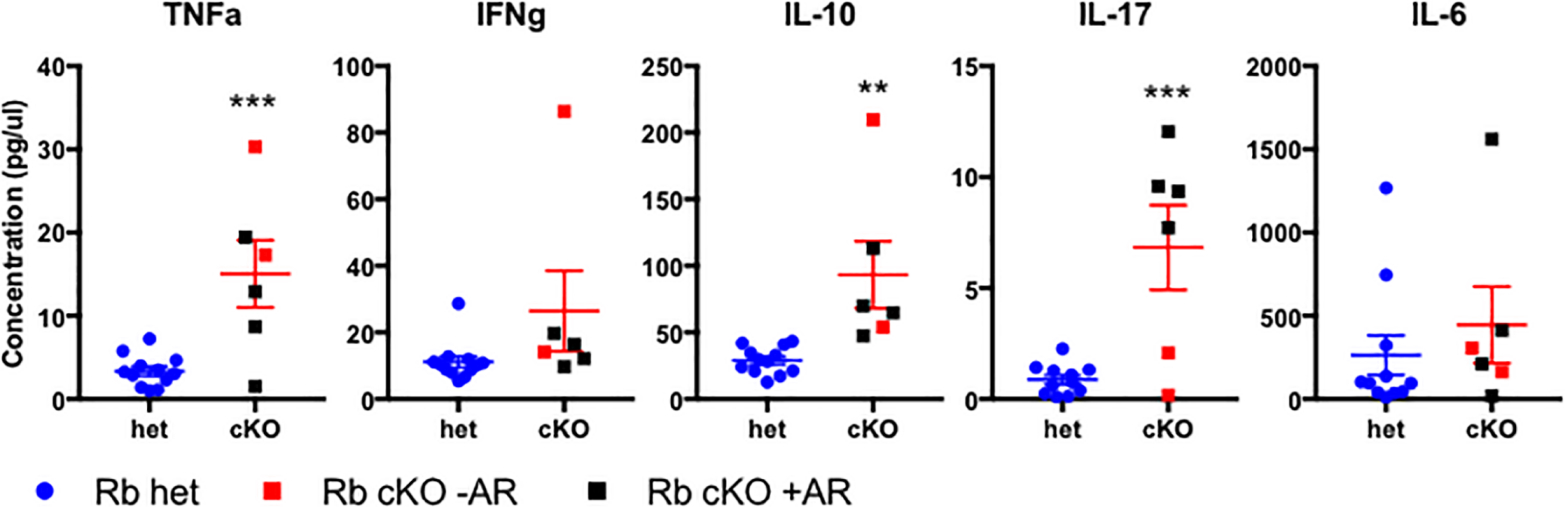
pRb loss in Tie2 lineage increases circulating cytokines in aged mice. ELISA array was used to measure circulating cytokines in serum of pRb het and cKO mice. Serum was isolated following overnight fast by cardiac puncture immediately after sacrifice. Mean ± SEM. P-value from Student’s T-Test, corrected for multiple measures by Holm-Sidak method (** < 0.005; ***<0.0005. N = 12 (het), 6 (cKO).

Because inflammation is a key driver of the early stages of aortic valve [22] disease we asked whether systemic inflammation was increased in pRb cKO mice. Tie2-cre drives recombination in hematopoietic stem cells [13], and conditional knockout of pRb in hematopoietic cells causes myeloproliferative disease [39]. pRb cKO mice were found to have splenomegaly, bone marrow cellular expansion, and a relative increase in the bone marrow monocyte population (S5 Fig). Since monocytes have been described in early CAVD lesions and secrete pro-inflammatory cytokines [22], we asked if markers of systemic inflammation were increased in pRb cKO mice using a cytokine ELISA array. TNFα, IL-10, and IL-17 were elevated in aged pRb cKO mice, compared to controls (Fig 6). Interestingly, IL-17 was only elevated in cKO mice with aortic regurgitation (black symbols). The presence of elevated circulating cytokines suggests that inflammation present in the cKO mice may be contributing to aortic valve pathology and valve dysfunction.

## Discussion

In this report, we demonstrate that conditional pRb loss in the Tie2 lineage is sufficient to cause age-dependent aortic valve thickening, ECM disorganization and regurgitation reminiscent of human fibrotic valvular disease or aortic valve sclerosis, a stage that precedes frank CAVD [3]. Our model differs from several other mouse models of aortic valve disease, where dysfunction is caused by defective embryonic valve development or aortic valve regurgitation secondary to aortic root dilation and leaflet prolapse; however, it is similar to existing models in that the primary outcome is aortic insufficiency and not calcification, likely due to the hemodynamic differences between mouse and man [42, 43]. pRb cKO pups were weaned at an expected Mendelian ratio and were found to have normal valve histology at 2 months of age and normal valve function at 6 months, arguing against a developmental effect. Instead, pRb cKO mice develop aortic valve pathology and dysregulation after 10–12 months of age without dilation of the aorta. Thus, our model recapitulates features of age-dependent aortic valve dysfunction including regurgitation commonly observed in humans. Intriguingly, two members of a human family carrying a retinoblastoma gene mutation have been found to have a bicuspid aortic valve potentially strengthening the importance of RB1 in aortic valve disease [44].

When healthy aortic valves transition to diseased valves, the resident VICs transition from a quiescent fibroblast phenotype to a state of activation resembling myofibroblast-like cells, which express mediators that can promote tissue remodeling. Similarly, the most striking molecular difference between pRb deficient and control valves was increased α-SMA expression throughout the leaflets of all aged pRb cKO valves, consistent with activation of pRb-deficient VICs into myofibroblasts. Because α-SMA expression was increased in all cKO valves, even when aortic valve regurgitation was not present, this finding is consistent with pRb being necessary to prevent the state of VIC activation that precedes ECM remodeling and valve dysfunction in our model.

A prevailing hypothesis in the field is that pathogenesis of aortic valve disease results from dysregulation of the regulatory pathways that control late embryonic valve development [45, 46]. Consistent with this theory, the reduced cellular density without changes in proliferation index within the aortic valve leaflets of pRb cKO mice is reminiscent of aortic valve remodeling during development [21]. Given the role pRb plays in regulation of terminal differentiation of several mesenchymal lineages, our finding in aortic valve leaflets supports a role for pRb in promoting VIC differentiation and quiescence. This model suggests a cell autonomous role for pRb in regulation of VIC differentiation that is required to prevent age-dependent aortic valve dysfunction.

We speculate on two factors that we consider to be candidates for modulating the transition of VICs to myofibroblasts in a pRb-dependent manner: Runx family transcription factors and TGFβ. Both of these factors have been directly linked to development of the myofibroblast in diseases states [47], as happens in aortic valve disease and in our model. Importantly, pRb and its regulators, the D-type cyclins and cyclin-dependent kinases (cdk), are well established to (a) directly influence Runx transcription factor function [48] and (b) mediate the effects of TGFβ [49]. In the latter case, it is intriguing to speculate that TGFβ effects in cells in which pRb function is lost genetically as in our model or through physiological inactivation by cdk activity as may occur in diseased tissue may switch from proliferation-suppressive (mediated by functional pRb) to favor the transition to the myofibroblast.

In addition to a VIC cell-autonomous role for pRb in aortic valve disease our results cannot exclude a non-cell autonomous mechanism. We asked whether the loss of pRb in hematopoietic cells derived from the Tie2 lineage affected the immune system in pRb cKO mice and whether there was increased inflammation, a known contributor to CAVD [50]. Several cytokines relevant to aortic valve disease were found to be increased in pRb cKO mice, including TNFα and IL-17. TNFα in particular is a pro-inflammatory cytokine that promotes CAVD progression [51]. The pro-inflammatory cytokine IL-17, which was specifically elevated in cKO mice with AR, is released from helper T-cells and functions to recruit monocytes into tissues [52]. While IL-17 has not been reported in association with CAVD, T-cell infiltration has been described in early lesions [53], and a population of gamma/delta T-cells resident in the AoV and aortic root that secrete IL-17 has been reported [54, 55]. Although T-cell and monocyte infiltration in our model was not obvious by H&E staining of pRb cKO valves, additional studies are required to fully delineate the role of the immune system in pRb cKO mice with aortic valve disease. Nevertheless, increased circulating cytokine levels support the concept that inflammation may be a valve-extrinsic factor contributing to the aortic valve abnormalities in our model.

Histological sections showed ECM remodeling in AoV leaflets of pRb cKO animals with regurgitation, and more detailed, micron scale and protein level observations revealed substantial differences. Using SHG imaging, a sensitive technique for measuring differences in collagen architecture that does not require tissue destruction [56], we observed increased collagen fiber variance and intensity in pRb cKO valve leaflets indicating a greater degree of ECM disorganization, which is characteristic of aortic valve disease [57]. Additionally, pRb cKO valves had a higher measured Young’s Modulus than controls, indicating further mechanical changes occurring in the diseased valve leaflets. A large body of literature has shown that increased substrate stiffness influences myofibroblast [58] and osteoblast differentiation [59]. Interestingly, pRb phosphorylation is inhibited at physiologic tissue stiffness and induced in pathological stiffness *in vitro*, suggesting that pathologic matrix remodeling itself can regulate cell cycling and differentiation via the pRb pathway [60].

The loss of tight, parallel collagen fibers has been shown to contribute to aortic valve dysfunction and provide nucleation sites for calcification [22, 61]. We observed differences in protein composition between pRb cKO and control mice that clustered with leaflet stiffness and collagen variance. Collagen I, a major component of the overall matrix structure [62], correlated with leaflet stiffness [63] and collagen fiber alignment was associated with fibrillins [64] and collagen XV [65], a known regulator of ECM organization [66]. The compilation of individual spectral counts showed a statistical difference in collagen VI expression between the pRb cKO and control mice. Collagen VI plays an important role in valve morphogenesis and valve development so may contribute to valve pathology [67]. Evidence from the cancer field demonstrates that pRb negatively regulates MMP activity [68], suggesting a mechanistic link between pRb loss/inactivation and increases in ECM remodeling in the aortic valve. Taken together, these data suggest that the pathological remodeling observed in pRb cKO valves is consistent with early stages of human fibrocalcific aortic valve disease. We can only speculate whether frank valve calcification might have been present in even older pRb cKO mice.

### Limitations to present study

In this report, we present a novel mouse model of aortic valve dysfunction; however, we acknowledge some limitations to this model. Because the Tie2-Cre driver is expected to cause recombination in cells other than VICs, we cannot exclude the possibility that our findings may be caused by the loss of pRb outside of the AoV. For example, using the Tie2-Cre driver does not allow us to rule out the unique contributions of circulating endothelial and CD45+ cells, which are also in the Tie2 lineage [13, 69] and accumulate in the AoV in an age-dependent manner [12, 70]. Alternative Cre drivers, such as Periostin [71] or Nfatc1 [72], that target VICs at different developmental time points, including cells not derived from the Tie2 lineage, may further clarify the contributions of *RB1* to aortic valve disease.

### Conclusion

Taken together, the pRb cKO mouse represents a model of age-dependent aortic valve regurgitation characterized by leaflet pathology, namely sclerosis, and circulating biomarkers of inflammation.

## Supporting information

S1 Fig Survival of pRb het and cKO mice. n = 21 (het) and 15 (cKO). ** Mantel-Cox P-value = 0.004.
(TIFF)

S2 Fig Histological analysis of aortic valve leaflet calcification in aged mice. Representative images of A) Alizarin Red] and B) Von Kossa stains demonstrate the lack of calcification in pRb het or cKO animals.
(TIFF)

S3 Fig Histological analysis of aortic valve leaflets from 2-month-old mice. A) Masson’s Trichrome, B) Movat pentachrome, and C) α-SMA Immunohistochemistry demonstrating the lack of distinct alteration in leaflets of 2 month old mice without pRb. Scale bar is 50μm.
(TIFF)

S4 Fig Differences in ECM protein components between pRb het and pRb cKO mice from proteomics analysis. Normalized spectral counts of structural ECM proteins collected through LC-MS/MS analysis of pRb het (blue) and pRb cKO (red) aortic valve leaflets (n = 3 for both conditions). Mean ± Std Dev; P-value (*p<0.05) from student’s t-test, corrected for multiple measures by Holm-Sidak method.
(TIFF)

S5 Fig Splenomegaly and bone marrow alterations in pRb cKO mice. A) Representative photo of spleens harvested from aged pRb het and cKO mice. B) Spleen weight normalized to tibia length, showing significantly splenomegaly in cKO mice (n = 15 het, 11 cKO; Student’s t-test p<0.0001). C) H&E staining of spleens from aged mice, showing increased white pulp in cKO samples as well as loss of typical nodular tissue structure, as in het spleens. D) H&E staining of the distal end of the tibia of aged mice, showing increased BM cells and reduced adipocytes in cKO animals. E) Representative FACS analysis of bone marrow, demonstrating increase of monocytes in cKO mice, as determined by cell surface expression Ly6C.
(TIFF)
