# Supplementary figures and images for: Conditional deletion of RB1 in the Tie2 lineage leads to aortic valve regurgitation
Source: PLoS One. 2018 Jan 5;13(1):e0190623. doi: 10.1371/journal.pone.0190623 (PMC5755794; doi:10.1371/journal.pone.0190623)

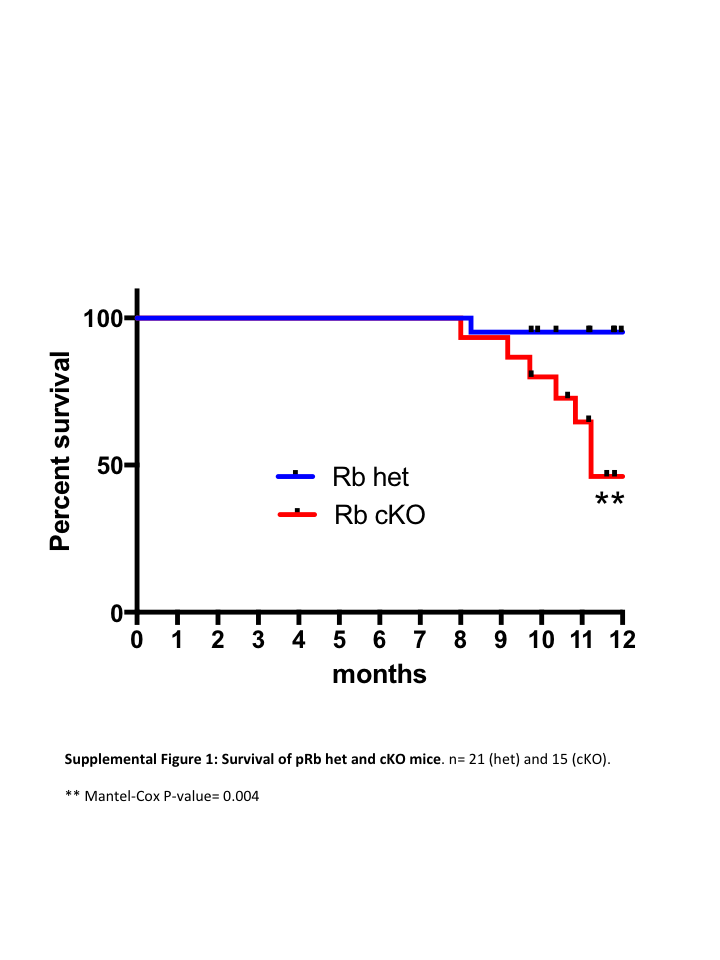

Supplement: S1 Fig — n = 21 (het) and 15 (cKO). ** Mantel-Cox P-value = 0.004. (TIFF) [file pone.0190623.s001.tiff]

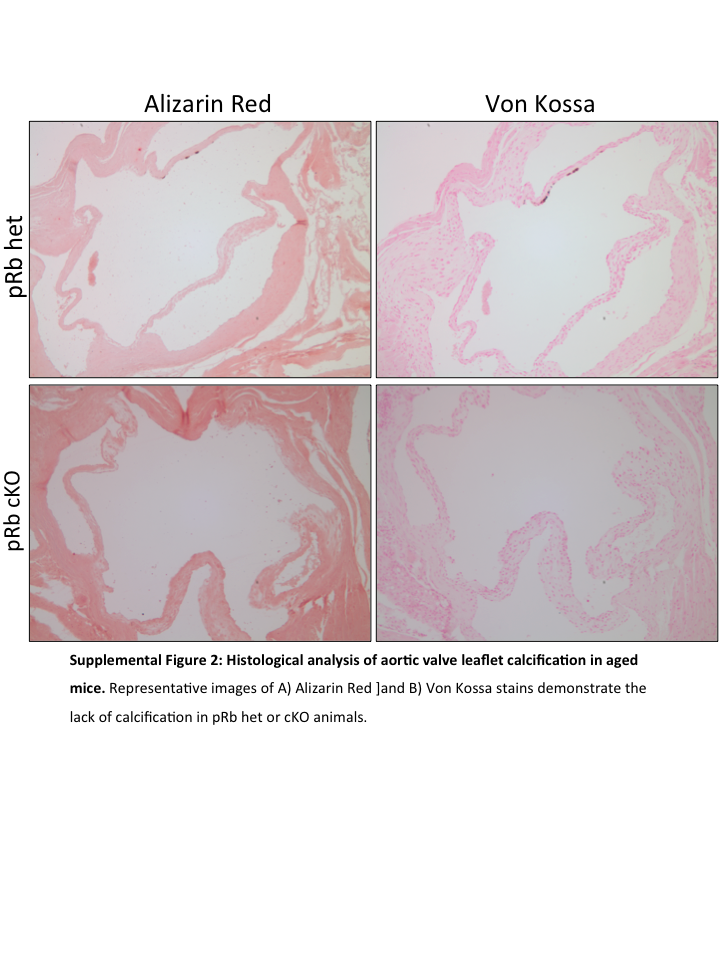

Supplement: S2 Fig — Representative images of A) Alizarin Red] and B) Von Kossa stains demonstrate the lack of calcification in pRb het or cKO animals. (TIFF) [file pone.0190623.s002.tiff]

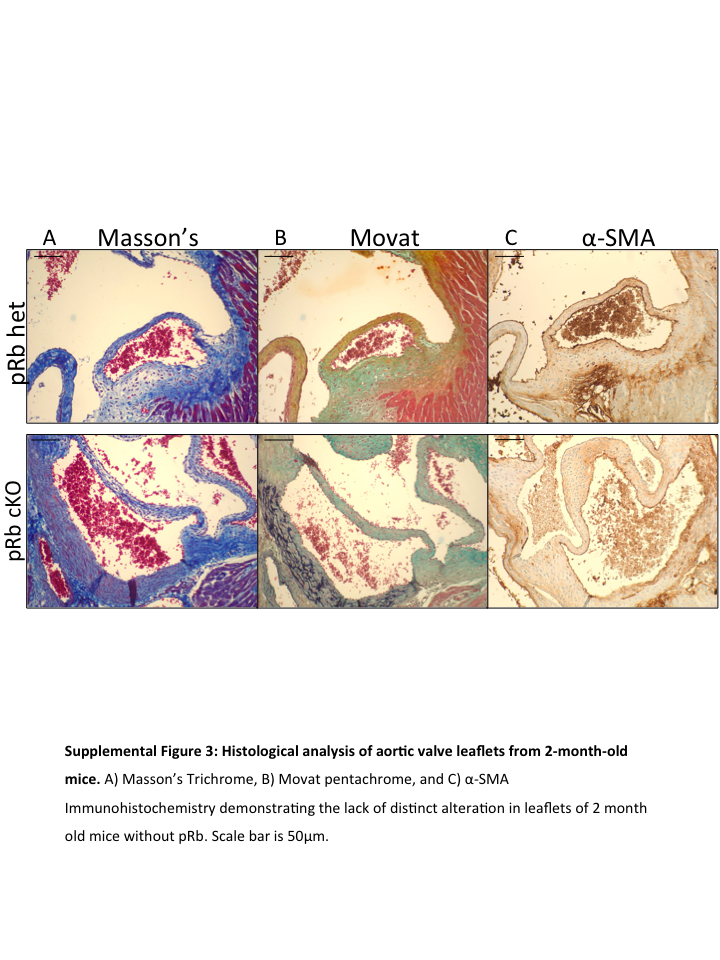

Supplement: S3 Fig — A) Masson’s Trichrome, B) Movat pentachrome, and C) α-SMA Immunohistochemistry demonstrating the lack of distinct alteration in leaflets of 2 month old mice without pRb. Scale bar is 50μm. (TIFF) [file pone.0190623.s003.tiff]

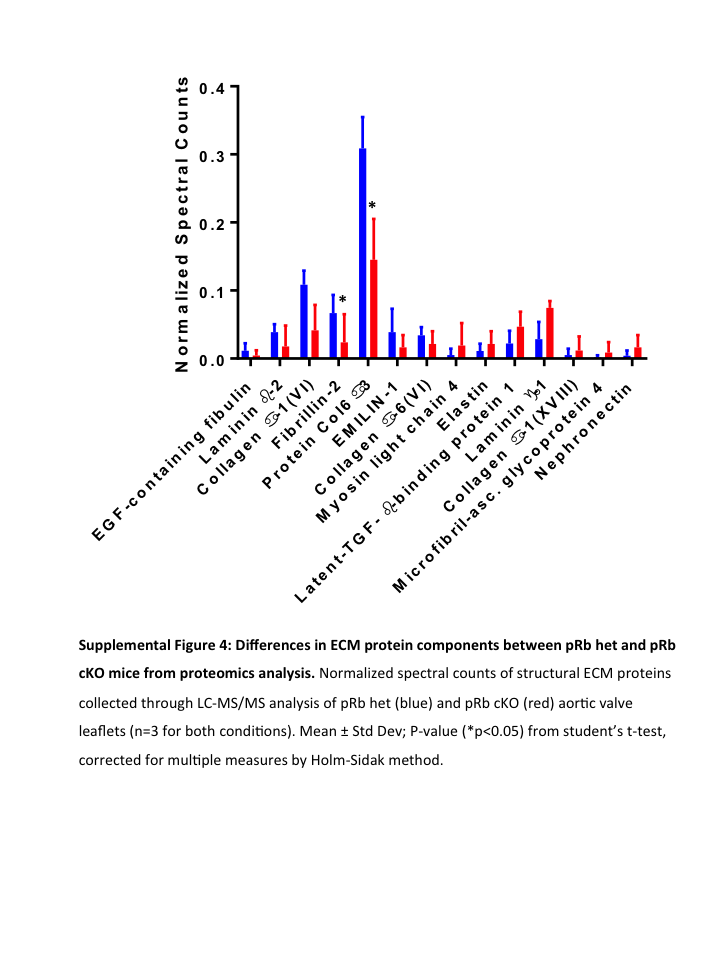

Supplement: S4 Fig — Normalized spectral counts of structural ECM proteins collected through LC-MS/MS analysis of pRb het (blue) and pRb cKO (red) aortic valve leaflets (n = 3 for both conditions). Mean ± Std Dev; P-value (*p<0.05) from student’s t-test, corrected for multiple measures by Holm-Sidak method. (TIFF) [file pone.0190623.s004.tiff]

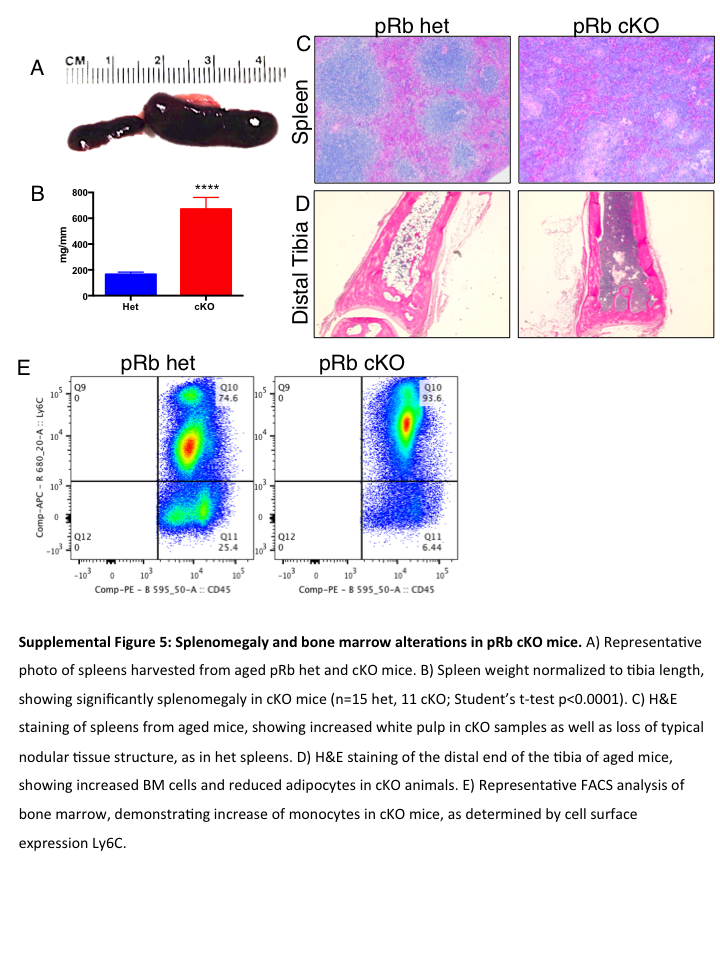

Supplement: S5 Fig — A) Representative photo of spleens harvested from aged pRb het and cKO mice. B) Spleen weight normalized to tibia length, showing significantly splenomegaly in cKO mice (n = 15 het, 11 cKO; Student’s t-test p<0.0001). C) H&E staining of spleens from aged mice, showing increased white pulp in cKO samples as well as loss of typical nodular tissue structure, as in het spleens. D) H&E staining of the distal end of the tibia of aged mice, showing increased BM cells and reduced adipocytes in cKO animals. E) Representative FACS analysis of bone marrow, demonstrating increase of monocytes in cKO mice, as determined by cell surface expression Ly6C. (TIFF) [file pone.0190623.s005.tiff]
